# Supplementary material for: Effects of oxytocin receptor agonism on acquisition and expression of pair bonding in male prairie voles
Source: Transl Psychiatry. 2024 Jul 15;14:286. doi: 10.1038/s41398-024-02993-x (PMC11251033; doi:10.1038/s41398-024-02993-x)
Supplement: Supplementary file 1 — Supplementary Material [file 41398_2024_2993_MOESM1_ESM.pdf]

Supplementary figures for manuscript

EFFECTS OF OXYTOCIN RECEPTOR AGONISM ON ACQUISITION AND EXPRESSION OF PAIR-BONDING IN MALE PRAIRIE VOLES

by

Michael C Johnson, Jonathan A Zweig, Yangmiao Zhang, Louis Nunez, Olga P Ryabinina, Marcel Hibert, Andrey E Ryabinin

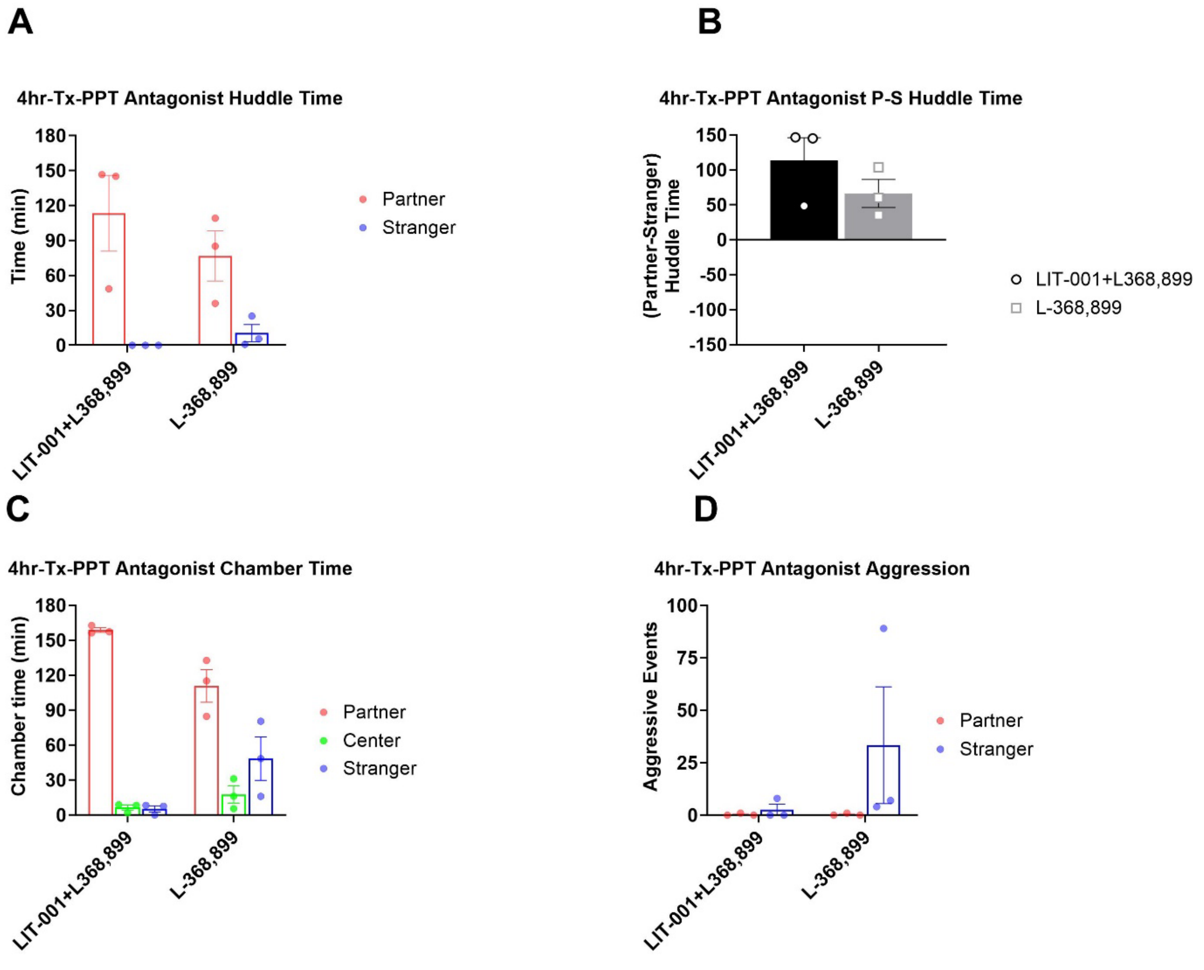

Fig S1. Analysis of male prairie voles that received LIT (10 mg/kg, n=3) and L-368,899 (10 mg/kg, n=3) after a 4-hour cohabitation and prior to 3-hour partner preference test. A. Total huddling time. B. Preferential huddling. C. Time spent in respective chambers by male prairie voles. D. Aggressive events. All bars reflect mean ± SEM. A, C, and D Kruskal-Wallis test followed by a post-hoc Mann-Whitney analysis, B One-Sample Wilcoxon Signed Rank Test.

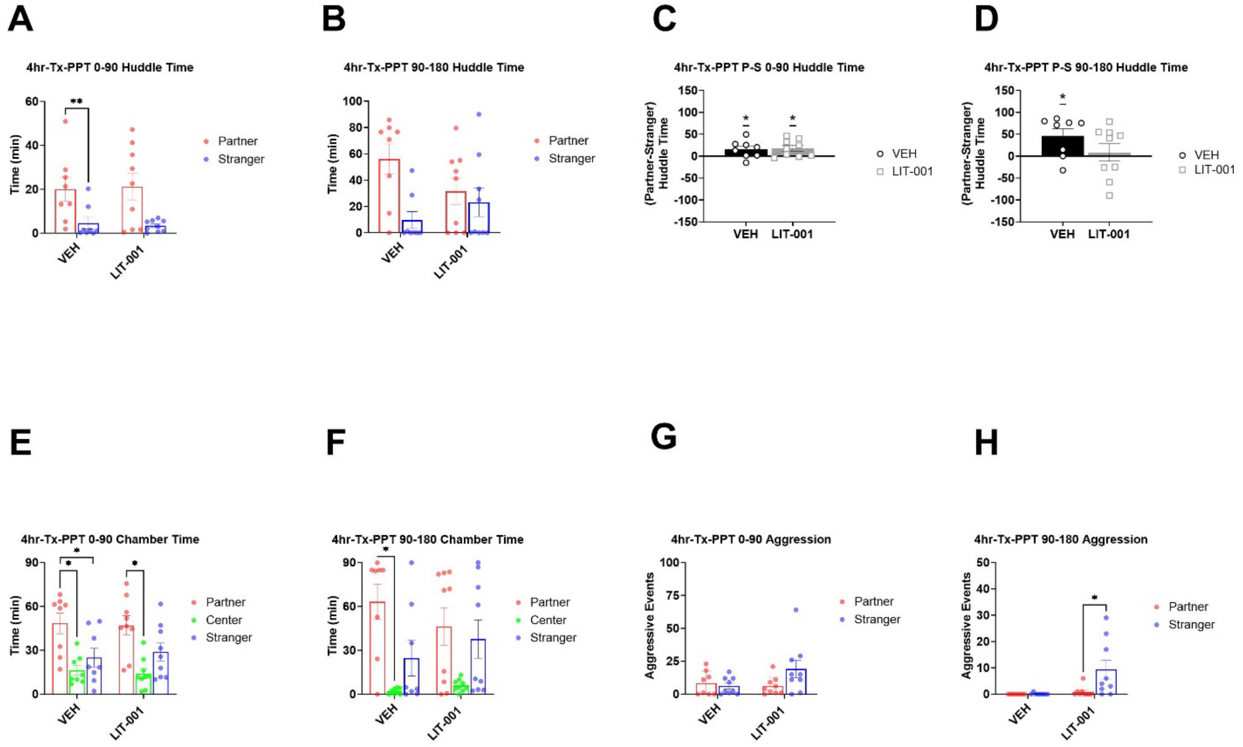

Fig S2. Temporal analysis of male prairie voles that received LIT (10 mg/kg, n=9) or vehicle (n=8) after a 4-hour cohabitation and prior to a 3-hour partner preference test. A. Aggressive events during the first half of the PPT. B. Aggressive events during the second half of the PPT. C. Preferential huddling during the first half of the PPT. D. Preferential huddling during the second half of the PPT. E. Time spent in respective chambers by male prairie voles during the first half of the PPT. F. Time spent in respective chambers by male prairie voles during the second half of the PPT. G. Aggressive events during the first half of the PPT. H. Aggressive events during the second half of the PPT. All bars reflect mean ± SEM. *, $p<0.05$; **, $p<0.01$, A, B, E, F, G and H Kruskal-Wallis test followed by a post-hoc Mann-Whitney analysis, C and D One-Sample Wilcoxon Signed Rank Test.

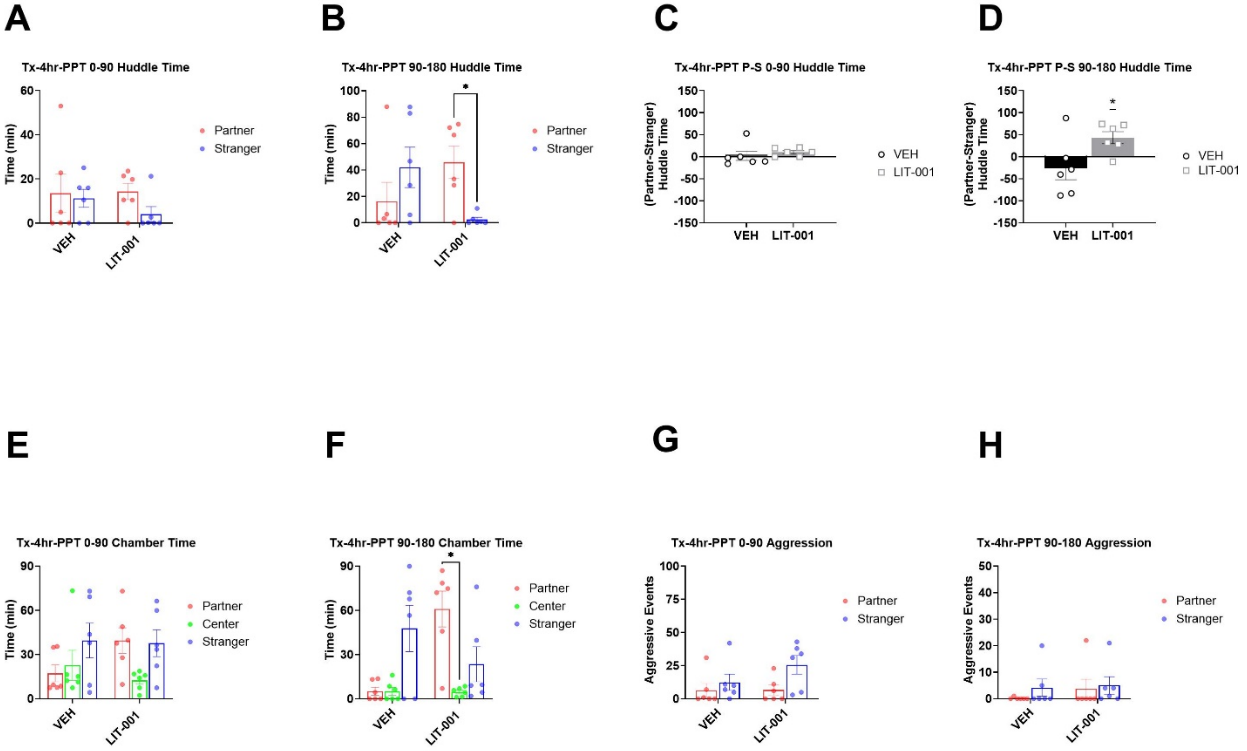

Fig S3. Temporal analysis of male prairie voles that received (10 mg/kg, n=6) or vehicle (n=6) prior to a 4-hour cohabitation followed by a 3-hour partner preference test. A. Aggressive events during the first half of the PPT. B. Aggressive events during the second half of the PPT. C. Preferential huddling during the first half of the PPT. D. Preferential huddling during the second half of the PPT. E. Time spent in respective chambers by male prairie voles during the first half of the PPT. F. Time spent in respective chambers by male prairie voles during the second half of the PPT. G. Aggressive events during the first half of the PPT. H. Aggressive events during the second half of the PPT. All bars reflect mean ± SEM. *, $p<0.05$, A, B, E, F, G and H Kruskal-Wallis test followed by a post-hoc Mann-Whitney analysis, C and D One-Sample Wilcoxon Signed Rank Test.

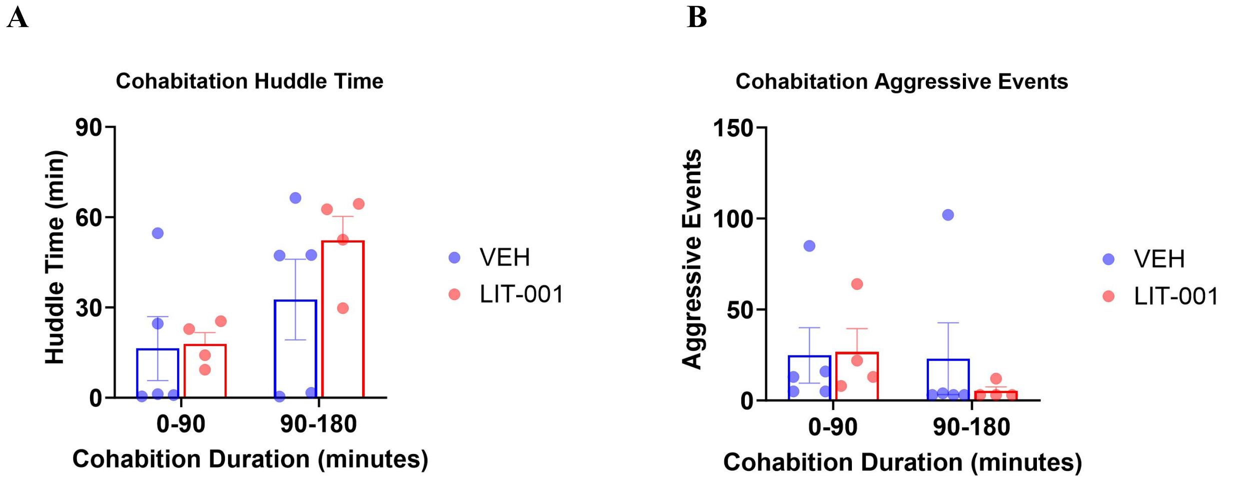

Fig S4. A. Huddling time and B. Aggressive events in male-female pairs during first 3 hours of a 4-hour cohabitation period after the male prairie vole received LIT (10 mg/kg, n=4) or vehicle (n=5). Bars reflect mean ± SEM of respective behavioral measures. There were no statistically significant differences between groups.

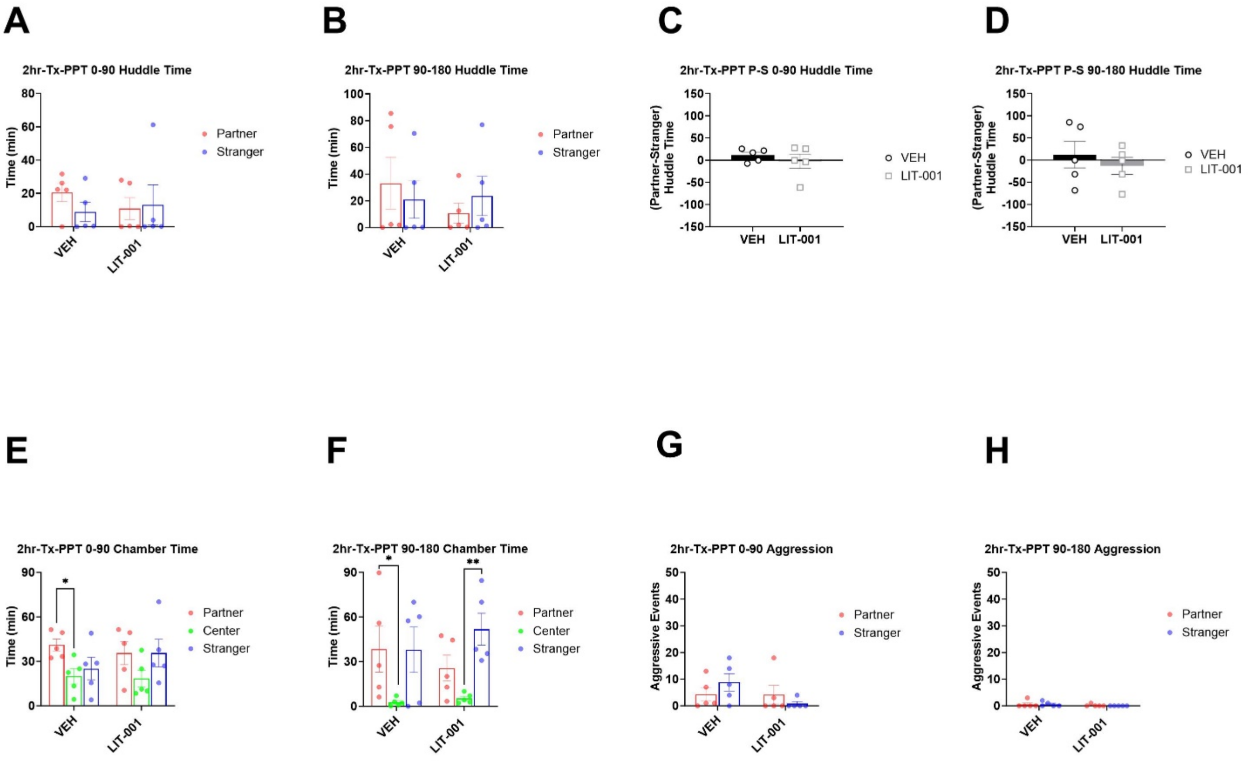

Fig S5. Temporal analysis of male prairie voles that received LIT (10 mg/kg, n=5) or vehicle (n=5) after a 2-hour cohabitation and prior to a 3-hour partner preference test. A. Aggressive events during the first half of the PPT. B. Aggressive events during the second half of the PPT. C. Preferential huddling during the first half of the PPT. D. Preferential huddling during the second half of the PPT. E. Time spent in respective chambers by male prairie voles during the first half of the PPT. F. Time spent in respective chambers by male prairie voles during the second half of the PPT. G. Aggressive events during the first half of the PPT. H. Aggressive events during the second half of the PPT. All bars reflect mean ± SEM. *, $p<0.05$; **, $p<0.01$, A, B, E, F, G and H Kruskal-Wallis test followed by a post-hoc Mann-Whitney analysis, B One-Sample Wilcoxon Signed Rank Test.
