# Supplementary material for: Effects of oxytocin receptor agonism on acquisition and expression of pair bonding in male prairie voles
Source: Transl Psychiatry. 2024 Jul 15;14:286. doi: 10.1038/s41398-024-02993-x (PMC11251033; doi:10.1038/s41398-024-02993-x)
Supplement: Supplementary file 2 — Supplementary Statistics Table [file 41398_2024_2993_MOESM2_ESM.pdf]

Statistics table for manuscript **EFFECTS OF OXYTOCIN RECEPTOR AGONISM ON EXPRESSION AND ACQUISITION OF PAIR-BONDING IN MALE PRAIRIE VOLES**			
Figure and Panel	Dependent Variable	Statistical Test Used	Statistical Report
F2A – 4hr-Tx-PPT	Huddle Time	Kruskal-Wallis test	$H(3) = 11.58$, $p = 0.009$
F2A – 4hr-Tx-PPT	Huddle Time	Mann-Whitney U test, comparison: Partner Animal vs. Stranger Animal, in VEH treated males	Partner Animal: M = 76.03, ±SEM = 15.10; Stranger Animal: M = 14.27, ±SEM = 8.47 $U(N_{\text{Partner}} = 8, N_{\text{Stranger}} = 8) = 5.0$, $p = 0.003$
F2A – 4hr-Tx-PPT	Huddle Time	Mann-Whitney U test, comparison: Partner Animal vs. Stranger Animal, in LIT-001 treated males	Partner Animal: M = 52.76, ±SEM = 13.80; Stranger Animal: M = 26.58, ±SEM = 11.28 $U(N_{\text{Partner}} = 9, N_{\text{Stranger}} = 9) = 25$, $p = 0.19$
F2B – 4hr-Tx-PPT	Partner-Stranger Huddle Time, VEH treated males	One-Sample Wilcoxon Signed Rank Test	N = 8, Hypothesized med.= 0, W = 33.0, Z = 2.10, $p = 0.036$
F2B – 4hr-Tx-PPT	Partner-Stranger Huddle Time, LIT treated males	One-Sample Wilcoxon Signed Rank Test	N = 9, Hypothesized med.= 0, W = 33.0, Z= 1.24, $p = 0.21$
F2C – 4hr-Tx-PPT	Chamber Time	Kruskal-Wallis test	$H(5) = 21.69$, $p = 0.0006$
F2C – 4hr-Tx-PPT	Chamber Time	Mann-Whitney U test, comparison: Partner Chamber vs. Center Chamber, in VEH treated males	Partner Chamber: M =111.66, ±SEM =18.16; Center Chamber: M =18.36, ±SEM = 3.33 $U(N_{\text{Partner}} = 8, N_{\text{Center}} = 8) = 4.0$, $p = 0.0018$
F2C – 4hr-Tx-PPT	Chamber Time	Mann-Whitney U test, comparison: Stranger Chamber vs. Center Chamber, in VEH treated males	Stranger Chamber: M = 49.64, ±SEM = 16.74; Center Chamber: M = 18.36, ±SEM = 3.33 $U(N_{\text{Stranger}} = 8, N_{\text{Center}} = 8) = 21$, $p = 0.27$
F2C – 4hr-Tx-PPT	Chamber Time	Mann-Whitney U test, comparison: Partner Chamber vs. Stranger Chamber, in VEH treated males	Partner Chamber: M = 111.66, ±SEM = 18.16; Stranger Chamber: M = 49.64, ±SEM = 16.74 $U(N_{\text{Partner}} = 8, N_{\text{Stranger}} = 8) = 11$, $p = 0.028$
F2C – 4hr-Tx-PPT	Chamber Time	Mann-Whitney U test, comparison: Partner Chamber vs. Center Chamber, in LIT treated males	Partner Chamber: M = 93.36, ±SEM = 18.16; Center Chamber: M = 20.06, ±SEM = 3.64

			$U(N_{Partner} = 9, N_{Center} = 9) = 5.0$, $p = 0.00078$
F2C – 4hr-Tx-PPT	Chamber Time	Mann-Whitney U test, comparison: Stranger Chamber vs. Center Chamber, in LIT treated males	Stranger Chamber: $M = 66.49$, $\pm SEM = 16.74$; Center Chamber: $M = 20.06$, $\pm SEM = 3.64$ $U(N_{Stranger} = 9, N_{Center} = 9) = 16$, $p = 0.031$
F2C – 4hr-Tx-PPT	Chamber Time	Mann-Whitney U test, comparison: Partner Chamber vs. Stranger Chamber, in LIT treated males	Partner Chamber: $M = 93.36$, $\pm SEM = 18.16$; Stranger Chamber: $M = 66.49$, $\pm SEM = 16.74$ $U(N_{Partner} = 9, N_{Stranger} = 9) = 23$, $p = 0.13$
F2D – 4hr-Tx-PPT	Aggressive Events	Kruskal-Wallis test	$H(3) = 5.68$, $p = 0.12$
F3A – Tx-4hr-PPT	Huddle Time	Kruskal-Wallis test	$H(3) = 4.22$, $p = 0.23$
F3A – Tx-4hr-PPT	Huddle Time	Mann-Whitney U test, comparison: Partner Animal vs. Stranger Animal, in VEH treated males	Partner Animal: $M = 31.69$, $\pm SEM = 22.08$; Stranger Animal: $M = 47.40$, $\pm SEM = 20.73$ $U(N_{Partner} = 6, N_{Stranger} = 6) = 17.0$, $p = 0.93$
F3A – Tx-4hr-PPT	Huddle Time	Mann-Whitney U test, comparison: Partner Animal vs. Stranger Animal, in LIT-001 treated males	Partner Animal: $M = 60.12$, $\pm SEM = 14.75$; Stranger Animal: $M = 6.52$, $\pm SEM = 3.92$ $U(N_{Partner} = 6, N_{Stranger} = 6) = 5.5$, $p = 0.043$
F3B – Tx-4hr-PPT	Partner-Stranger Huddle Time, VEH treated males	One-Sample Wilcoxon Signed Rank Test	N = 6, Hypothesized med.= 0, W = 8.0, Z= -0.52, $p = 0.60$
F3B – Tx-4hr-PPT	Partner-Stranger Huddle Time, LIT treated males	One-Sample Wilcoxon Signed Rank Test	N = 6, Hypothesized med.= 0, W = 20.0, Z= 1.99, $p = 0.046$
F3C – Tx-4hr-PPT	Chamber Time	Kruskal-Wallis test	$H(5) = 13.51$, $p = 0.19$
F3C – Tx-4hr-PPT	Chamber Time	Mann-Whitney U test, comparison: Partner Chamber vs. Center Chamber, in VEH treated males	Partner Chamber: $M = 22.42$, $\pm SEM = 6.65$; Center Chamber: $M = 27.82$, $\pm SEM = 12.39$ $U(N_{Partner} = 6, N_{Center} = 6) = 15$, $p = 0.69$
F3C – Tx-4hr-PPT	Chamber Time	Mann-Whitney U test, comparison: Stranger Chamber vs. Center Chamber, in VEH treated males	Stranger Chamber: $M = 87.28$, $\pm SEM = 21.21$; Center Chamber: $M = 27.82$, $\pm SEM = 12.39$

			$U(N_{Stranger} = 6, N_{Center} = 6) = 8.0$, $p = 0.13$
F3C – Tx-4hr-PPT	Chamber Time	Mann-Whitney U test, comparison: Partner Chamber vs. Stranger Chamber, in VEH treated males	Partner Chamber: M = 22.42, ±SEM = 6.65; Stranger Chamber: M = 87.28, ±SEM = 21.21 $U(N_{Partner} = 6, N_{Stranger} = 6) = 6.0$, $p = 0.064$
F3C – Tx-4hr-PPT	Chamber Time	Mann-Whitney U test, comparison: Partner Chamber vs. Center Chamber, in LIT treated males	Partner Chamber: M = 22.42, ±SEM = 6.65; Center Chamber: M = 17.18, ±SEM = 2.27 $U(N_{Partner} = 6, N_{Center} = 6) = 4.0$, $p = 0.025$
F3C – Tx-4hr-PPT	Chamber Time	Mann-Whitney U test, comparison: Stranger Chamber vs. Center Chamber, in LIT treated males	Stranger Chamber: M = 61.06, ±SEM = 20.34; Center Chamber: M = 17.18, ±SEM = 2.27 $U(N_{Stranger} = 6, N_{Center} = 6) = 5.0$, $p = 0.041$
F3C – Tx-4hr-PPT	Chamber Time	Mann-Whitney U test, comparison: Partner Chamber vs. Stranger Chamber, in LIT treated males	Partner Chamber: M = 100.36, ±SEM = 19.51; Stranger Chamber: M = 61.06, ±SEM = 20.34 $U(N_{Partner} = 6, N_{Stranger} = 6) = 10.0$, $p = 0.24$
F3D – Tx-4hr-PPT	Aggressive Events	Kruskal-Wallis test	$H(3) = 5.82$, $p = 0.12$
F4A – 2hr-Tx-PPT	Huddle Time	Kruskal-Wallis test	$H(3) = 1.08$, $p = 0.78$
F4A – 2hr-Tx-PPT	Huddle Time	Mann-Whitney U test, comparison: Partner Animal vs. Stranger Animal, in VEH treated males	Partner Animal: M = 53.57, ±SEM = 20.97; Stranger Animal: M = 29.83, ±SEM = 15.47 $U(N_{Partner} = 5, N_{Stranger} = 5) = 9.0$, $p = 0.54$
F4A – 2hr-Tx-PPT	Huddle Time	Mann-Whitney U test, comparison: Partner Animal vs. Stranger Animal, in LIT-001 treated males	Partner Animal: M = 21.68, ±SEM = 20.97; Stranger Animal: M = 36.71, ±SEM = 26.04 $U(N_{Partner} = 5, N_{Stranger} = 5) = 12.0$, $p > 0.99$
F4B – 2hr-Tx-PPT	Partner-Stranger Huddle Time, VEH treated males	One-Sample Wilcoxon Signed Rank Test	N = 5, Hypothesized med.= 0, W = 9.0, Z= 0.40 , $p = 0.68$

F4B – 2hr-Tx-PPT	Partner-Stranger Huddle Time, LIT treated males	One-Sample Wilcoxon Signed Rank Test	N = 5, Hypothesized med.= 0, W = 7.0, Z= -0.13 , *p* = 0.89
F4C – 2hr-Tx-PPT	Chamber Time	Kruskal-Wallis test	$H(5) = 13.44$, $p = 0.019$
F4C – 2hr-Tx-PPT	Chamber Time	Mann-Whitney U test, comparison: Partner Chamber vs. Center Chamber, in VEH treated males	Partner Chamber: 79.49, ±*SEM* = 17.38; Center Chamber: *M* = 22.66, ±*SEM* = 5.37 $U(N_{Partner} = 5, N_{Center} = 5) = 0$, $p = 0.0079$
F4C – 2hr-Tx-PPT	Chamber Time	Mann-Whitney U test, comparison: Stranger Chamber vs. Center Chamber, in VEH treated males	Stranger Chamber: *M* = 63.14, ±*SEM* = 16.28; Center Chamber: *M* = 22.66, ±*SEM* = 5.37 $U(N_{Stranger} = 5, N_{Center} = 5) = 5$, $p = 0.15$
F4C – 2hr-Tx-PPT	Chamber Time	Mann-Whitney U test, comparison: Partner Chamber vs. Stranger Chamber, in VEH treated males	Partner Chamber: *M* = 61.28, ±*SEM* = 17.38; Stranger Chamber: *M* = 63.14, ±*SEM* = 16.28 $U(N_{Partner} = 5, N_{Stranger} = 5) = 10$, $p = 0.69$
F4C – 2hr-Tx-PPT	Chamber Time	Mann-Whitney U test, comparison: Partner Chamber vs. Center Chamber, in LIT treated males	Partner Chamber: *M* = 61.28, ±*SEM* = 12.65; Center Chamber: *M* = 23.75, ±*SEM* = 7.07 $U(N_{Partner} = 5, N_{Center} = 5) = 4$, $p = 0.095$
F4C – 2hr-Tx-PPT	Chamber Time	Mann-Whitney U test, comparison: Stranger Chamber vs. Center Chamber, in LIT treated males	Stranger Chamber: *M* = 87.44, ±*SEM* = 17.49; Center Chamber: *M* = 23.75, ±*SEM* = 7.07 $U(N_{Stranger} = 5, N_{Center} = 5) = 0$, $p = 0.0079$
F4C – 2hr-Tx-PPT	Chamber Time	Mann-Whitney U test, comparison: Partner Chamber vs. Stranger Chamber, in LIT treated males	Partner Chamber: *M* = 61.28, ±*SEM* = 12.65; Stranger Chamber: *M* = 87.44, ±*SEM* = 17.49 $U(N_{Partner} = 5, N_{Stranger} = 5) = 9.0$, $p = 0.54$
F4D – 2hr-Tx-PPT	Aggressive Events	Kruskal-Wallis test	$H(3) = 5.07$, $p = 0.16$
S1A – 4hr-Tx-PPT Antagonist	Huddle Time	Kruskal-Wallis test	$H(3) = 9.80$, $p = 0.0006$
S1B – 4hr-Tx-PPT Antagonist	Partner-Stranger Huddle Time, LIT-001+L368,899	One-Sample Wilcoxon Signed Rank Test	N = 3, Hypothesized med.= 0, W = 6.0, Z= 1.60, *p* = 0.10

	treated males		
S1B – 4hr-Tx-PPT Antagonist	Partner-Stranger Huddle Time,<br>L-368,899<br>treated males	One-Sample Wilcoxon Signed Rank Test	N = 3, Hypothesized med.= 0, W = 6.0, Z= 1.60, *p* = 0.10
S1C – 4hr-Tx-PPT Antagonist	Chamber Time	Kruskal-Wallis test	$H(3) = 14.66$, $p = 0.012$
S1D – 4hr-Tx-PPT Antagonist	Aggressive Events	Kruskal-Wallis test	$H(3) = 5.41$, $p = 0.14$
S2A – 4hr-Tx-PPT 0-90	Huddle Time	Kruskal-Wallis test	$H(3) = 11.95$, $p = 0.0076$
S2A – 4hr-Tx-PPT 0-90	Huddle Time	Mann-Whitney U test, comparison: Partner Animal vs. Stranger Animal, in VEH treated males	Partner Animal: M = 20.05, ±SEM = 5.51; Stranger Animal: M = 4.58, ±SEM = 2.65 $U(N_{Partner} = 8, N_{Stranger} = 8) = 6.0$, $p = 0.005$
S2A – 4hr-Tx-PPT 0-90	Huddle Time	Mann-Whitney U test, comparison: Partner Animal vs. Stranger Animal, in LIT-001 treated males	Partner Animal: M = 21.21, ±SEM = 5.51; Stranger Animal: M = 3.36, ±SEM = 0.86 $U(N_{Partner} = 9, N_{Stranger} = 9) = 20$, $p = 0.077$
S2B – 4hr-Tx-PPT 90-180	Huddle Time	Kruskal-Wallis test	$H(3) = 6.89$, $p = 0.075$
S2B – 4hr-Tx-PPT 90-180	Huddle Time	Mann-Whitney U test, comparison: Partner Animal vs. Stranger Animal, in VEH treated males	Partner Animal: M = 55.97, ±SEM = 11.58; Stranger Animal: M = 9.67, ±SEM = 6.41 $U(N_{Partner} = 8, N_{Stranger} = 8) = 9.5$, $p = 0.015$
S2B – 4hr-Tx-PPT 90-180	Huddle Time	Mann-Whitney U test, comparison: Partner Animal vs. Stranger Animal, in LIT-001 treated males	Partner Animal: M = 31.54, ±SEM = 10.03; Stranger Animal: M = 23.21, ±SEM = 10.87 $U(N_{Partner} = 9, N_{Stranger} = 9) = 34$, $p = 0.60$
S2C – 4hr-Tx-PPT 0-90	Partner-Stranger Huddle Time, VEH treated males	One-Sample Wilcoxon Signed Rank Test	N = 8, Hypothesized med.= 0, W = 32.0, Z= 1.96, *p* = 0.05
S2C – 4hr-Tx-PPT 0-90	Partner-Stranger Huddle Time, LIT treated males	One-Sample Wilcoxon Signed Rank Test	N = 9, Hypothesized med.= 0, W = 40, Z= 2.07, *p* = 0.038
S2D – 4hr-Tx-PPT 90-180	Partner-Stranger Huddle Time, VEH treated males	One-Sample Wilcoxon Signed Rank Test	N = 8, Hypothesized med.= 0, W = 26.0, Z= 2.02, *p* = 0.043
S2D – 4hr-Tx-PPT 90-180	Partner-Stranger Huddle Time, LIT treated males	One-Sample Wilcoxon Signed Rank Test	N = 9, Hypothesized med.= 0, W = 26.0, Z= 0.41, *p* = 0.67
S2E – 4hr-Tx-PPT 0-90	Chamber Time	Kruskal-Wallis test	$H(5) = 21.000$, $p = 0.0008$

S2E – 4hr-Tx-PPT 0-90	Chamber Time	Mann-Whitney U test, comparison: Partner Chamber vs. Center Chamber, in VEH treated males	Partner Chamber: *M* = 48.42, ±*SEM* = 7.10; Center Chamber: *M* = 16.29, ±*SEM* = 3.45 $U(N_{Partner} = 8, N_{Center} = 8) = 5$, $p = 0.0029$
S2E – 4hr-Tx-PPT 0-90	Chamber Time	Mann-Whitney U test, comparison: Stranger Chamber vs. Center Chamber, in VEH treated males	Stranger Chamber: *M* = 24.99, ±*SEM* = 6.42; Center Chamber: *M* = 16.29, ±*SEM* = 3.45 $U(N_{Stranger} = 8, N_{Center} = 8) = 23$, $p = 0.38$
S2E – 4hr-Tx-PPT 0-90	Chamber Time	Mann-Whitney U test, comparison: Partner Chamber vs. Stranger Chamber, in VEH treated males	Partner Chamber: *M* = 48.42, ±*SEM* = 7.10; Stranger Chamber: *M* = 24.99, ±*SEM* = 6.42 $U(N_{Partner} = 8, N_{Stranger} = 8) = 11$, $p = 0.028$
S2E – 4hr-Tx-PPT 0-90	Chamber Time	Mann-Whitney U test, comparison: Partner Chamber vs. Center Chamber, in LIT treated males	Partner Chamber: *M* = 47.04, ±*SEM* = 6.53; Center Chamber: *M* = 14.05, ±*SEM* = 3.44 $U(N_{Partner} = 9, N_{Center} = 9) = 4.0$, $p = 0.00049$
S2E – 4hr-Tx-PPT 0-90	Chamber Time	Mann-Whitney U test, comparison: Stranger Chamber vs. Center Chamber, in LIT treated males	Stranger Chamber: *M* = 28.82, ±*SEM* = 6.11; Center Chamber: *M* = 14.05, ±*SEM* = 3.44 $U(N_{Stranger} = 9, N_{Center} = 9) = 22$, $p = 0.11$
S2E – 4hr-Tx-PPT 0-90	Chamber Time	Mann-Whitney U test, comparison: Partner Chamber vs. Stranger Chamber, in LIT treated males	Partner Chamber: *M* = 47.04, ±*SEM* = 6.53; Stranger Chamber: *M* = 28.82, ±*SEM* = 6.11 $U(N_{Partner} = 9, N_{Stranger} = 9) = 19$, $p = 0.062$
S2F – 4hr-Tx-PPT 90-180	Chamber Time	Kruskal-Wallis test	$H(5) = 15.29$, $p = 0.0092$
S2F – 4hr-Tx-PPT 90-180	Chamber Time	Mann-Whitney U test, comparison: Partner Chamber vs. Center Chamber, in VEH treated males	Partner Chamber: *M* =63.24, ±*SEM* = 12.10; Center Chamber: *M* = 2.07, ±*SEM* = 0.68 $U(N_{Partner} = 8, N_{Center} = 8) = 7$, $p = 0.0066$
S2F – 4hr-Tx-PPT 90-180	Chamber Time	Mann-Whitney U test, comparison: Stranger	Stranger Chamber: *M* = 24.65, ±*SEM* = 12.15;

		Chamber vs. Center Chamber, in VEH treated males	Center Chamber: *M* = 2.07, ±*SEM* = 0.68 $U(N_{Stranger}$ 8= , N_{Center} = 8) = 19, p = 0.19
S2F – 4hr-Tx-PPT 90-180	Chamber Time	Mann-Whitney U test, comparison: Partner Chamber vs. Stranger Chamber, in VEH treated males	Partner Chamber: *M* = 63.24, ±*SEM* = 12.10; Stranger Chamber: *M* = 24.65, ±*SEM* = 12.15 $U(N_{Partner}$ 8= , $N_{Stranger}$ = 8) = 16.5, p = 0.11
S2F – 4hr-Tx-PPT 90-180	Chamber Time	Mann-Whitney U test, comparison: Partner Chamber vs. Center Chamber, in LIT treated males	Partner Chamber: *M* = 46.31, ±*SEM* = 12.84; Center Chamber: *M* =6.00, ±*SEM* = 1.36 $U(N_{Partner}$ 9= , N_{Center} = 9) = 18.5, p = 0.051
S2F – 4hr-Tx-PPT 90-180	Chamber Time	Mann-Whitney U test, comparison: Stranger Chamber vs. Center Chamber, in LIT treated males	Stranger Chamber: *M* = 37.66, ±*SEM* = 13.00; Center Chamber: *M* = 6.00, ±*SEM* = 1.36 $U(N_{Stranger} = 9, N_{Center} = 9)$ = 24, p = 0.16
S2F – 4hr-Tx-PPT 90-180	Chamber Time	Mann-Whitney U test, comparison: Partner Chamber vs. Stranger Chamber, in LIT treated males	Partner Chamber: *M* = 46.31, ±*SEM* = 12.84; Stranger Chamber: *M* = 37.66, ±*SEM* = 13.00 $U(N_{Partner} = 9, N_{Stranger} = 9)$ = 40, $p > 0.99$
S2G – 4hr-Tx-PPT 0-90	Aggressive Events	Kruskal-Wallis test	$H(3) = 3.92$, $p = 0.26$
S2H – 4hr-Tx-PPT 90-180	Aggressive Events	Kruskal-Wallis test	$H(3) = 15.73$, $p = 0.0013$
S2H – 4hr-Tx-PPT 90-180	Aggressive Events	Mann-Whitney U test, comparison: Partner Animal vs. Stranger Animal, in VEH treated males	Partner Animal: *M* = 0.0, ±*SEM* = 0.0 ; Stranger Chamber: *M* = 0.12, ±*SEM* = 0.12 $U(N_{Partner} = 8, N_{Stranger} = 8)$ = 28, $p > 0.99$
S2H – 4hr-Tx-PPT 90-180	Aggressive Events	Mann-Whitney U test, comparison: Partner Animal vs. Stranger Animal, in LIT treated males	Partner Chamber: *M* = 0.88, ±*SEM* = 0.65; Stranger Chamber: *M* = 9.33, ±*SEM* = 3.61 $U(N_{Partner} = 9, N_{Stranger} = 9)$ = 15.5, p = 0.021
S3A – Tx-4hr-PPT 0-90	Huddle Time	Kruskal-Wallis test	$H(3) = 2.18$, $p = 0.53$
S3A – Tx-4hr-PPT -90	Huddle Time	Mann-Whitney U test, comparison: Partner	Partner Animal: *M* = 13.48, ±*SEM* = 8.68;

		Animal vs. Stranger Animal, in VEH treated males	Stranger Animal: *M* = 11.26, ±*SEM* = 4.03 $U(N_{Partner} = 6, N_{Stranger} = 6) = 18.0$, $p > 0.99$
S3A – Tx-4hr-PPT 0-90	Huddle Time	Mann-Whitney U test, comparison: Partner Animal vs. Stranger Animal, in LIT-001 treated males	Partner Animal: *M* = 14.28, ±*SEM* = 3.65; Stranger Animal: *M* = 4.02, ±*SEM* = 3.46 $U(N_{Partner} = 6, N_{Stranger} = 6) = 8.0$, $p = 0.12$
S3B – Tx-4hr-PPT 90-180	Huddle Time	Kruskal-Wallis test	$H(3) = 5.95$, $p = 0.11$
S3B – Tx-4hr-PPT 90-180	Huddle Time	Mann-Whitney U test, comparison: Partner Animal vs. Stranger Animal, in VEH treated males	Partner Animal: *M* = 16.29, ±*SEM* = 14.36; Stranger Animal: *M* = 41.96, ±*SEM* = 15.30 $U(N_{Partner} = 6, N_{Stranger} = 6) = 10.5$, $p = 0.25$
S3B – Tx-4hr-PPT 90-180	Huddle Time	Mann-Whitney U test, comparison: Partner Animal vs. Stranger Animal, in LIT-001 treated males	Partner Animal: *M* = 45.84, ±*SEM* = 12.26; Stranger Animal: *M* = 2.49, ±*SEM* = 1.75 $U(N_{Partner} = 6, N_{Stranger} = 6) = 5.0$, $p = 0.041$
S3C – Tx-4hr-PPT 0-90	Partner-Stranger Huddle Time, VEH treated males	One-Sample Wilcoxon Signed Rank Test	N = 6, Hypothesized med.= 0, W = 5.0, Z= -0.67, *p* = 0.5
S3C – Tx-4hr-PPT 0-90	Partner-Stranger Huddle Time, LIT treated males	One-Sample Wilcoxon Signed Rank Test	N = 6, Hypothesized med.= 0, W = 19.0, Z= 1.78, *p* = 0.075
S3D – Tx-4hr-PPT 90-180	Partner-Stranger Huddle Time, VEH treated males	One-Sample Wilcoxon Signed Rank Test	N = 6, Hypothesized med.= 0, W = 6.0, Z= -0.94, *p* = 0.34
S3D – Tx-4hr-PPT 90-180	Partner-Stranger Huddle Time, LIT treated males	One-Sample Wilcoxon Signed Rank Test	N = 6, Hypothesized med.= 0, W = 20.0, Z= 1.99, *p* = 0.046
S3E – Tx-4hr-PPT 0-90	Chamber Time	Kruskal-Wallis test	$H(5) = 8.42$, $p = 0.13$
S3E – Tx-4hr-PPT 0-90	Chamber Time	Mann-Whitney U test, comparison: Partner Chamber vs. Center Chamber, in VEH treated males	Partner Chamber: *M* =17.20, ±*SEM* = 5.69; Center Chamber: *M* = 22.63, ±*SEM* = 10.20 $U(N_{Partner} = 6, N_{Center} = 6) = 14.0$, $p = 0.58$
S3E – Tx-4hr-PPT 0-90	Chamber Time	Mann-Whitney U test, comparison: Stranger Chamber vs. Center Chamber, in VEH treated males	Stranger Chamber: *M* = 39.54, ±*SEM* = 11.81; Center Chamber: *M* = 22.63, ±*SEM* = 10.20

			$U(N_{Stranger} = 6, N_{Center} = 6) = 15.0$, $p = 0.69$
S3E – Tx-4hr-PPT 0-90	Chamber Time	Mann-Whitney U test, comparison: Partner Chamber vs. Stranger Chamber, in VEH treated males	Partner Chamber: $M = 17.20$, $\pm SEM = 5.69$; Stranger Chamber: $M = 39.54$, $\pm SEM = 11.81$ $U(N_{Partner} = 6, N_{Stranger} = 6) = 9.0$, $p = 0.17$
S3E – Tx-4hr-PPT 0-90	Chamber Time	Mann-Whitney U test, comparison: Partner Chamber vs. Center Chamber, in LIT treated males	Partner Chamber: $M = 39.48$, $\pm SEM = 8.64$; Center Chamber: $M = 12.48$, $\pm SEM = 2.60$ $U(N_{Partner} = 6, N_{Center} = 6) = 4.0$, $p = 0.025$
S3E – Tx-4hr-PPT 0-90	Chamber Time	Mann-Whitney U test, comparison: Stranger Chamber vs. Center Chamber, in LIT treated males	Stranger Chamber: $M = 37.62$, $\pm SEM = 9.09$; Center Chamber: $M = 12.48$, $\pm SEM = 2.60$ $U(N_{Stranger} = 6, N_{Center} = 6) = 4.0$, $p = 0.025$
S3E – Tx-4hr-PPT 0-90	Chamber Time	Mann-Whitney U test, comparison: Partner Chamber vs. Stranger Chamber, in LIT treated males	Partner Chamber: $M = 39.48$, $\pm SEM = 8.64$; Stranger Chamber: $M = 37.62$, $\pm SEM = 9.09$ $U(N_{Partner} = 6, N_{Stranger} = 6) = 15.0$, $p = 0.69$
S3F – Tx-4hr-PPT 90-180	Chamber Time	Kruskal-Wallis test	$H(5) = 12.97$, $p = 0.023$
S3F – Tx-4hr-PPT 90-180	Chamber Time	Mann-Whitney U test, comparison: Partner Chamber vs. Center Chamber, in VEH treated males	Partner Chamber: $M = 5.22$, $\pm SEM = 2.69$; Center Chamber: $M = 5.19$, $\pm SEM = 2.66$ $U(N_{Partner} = 6, N_{Center} = 6) = 17.5$, $p > 0.99$
S3F – Tx-4hr-PPT 90-180	Chamber Time	Mann-Whitney U test, comparison: Stranger Chamber vs. Center Chamber, in VEH treated males	Stranger Chamber: $M = 47.73$, $\pm SEM = 15.71$; Center Chamber: $M = 5.19$, $\pm SEM = 2.66$ $U(N_{Stranger} = 6, N_{Center} = 6) = 9.0$, $p = 0.16$
S3F – Tx-4hr-PPT 90-180	Chamber Time	Mann-Whitney U test, comparison: Partner Chamber vs. Stranger Chamber, in VEH treated males	Partner Chamber: $M = 5.22$, $\pm SEM = 2.69$; Stranger Chamber: $M = 5.19$, $\pm SEM = 2.66$ $U(N_{Partner} = 6, N_{Stranger} = 6) = 9.0$, $p = 0.16$

S3F – Tx-4hr-PPT 90-180	Chamber Time	Mann-Whitney U test, comparison: Partner Chamber vs. Center Chamber, in LIT treated males	Partner Chamber: *M* = 60.87, ±*SEM* = 12.15; Center Chamber: *M* = 4.69, ±*SEM* = 1.23 $U(N_{Partner} = 6, N_{Center} = 6) = 1.0$, $p = 0.004$
S3F – Tx-4hr-PPT 90-180	Chamber Time	Mann-Whitney U test, comparison: Stranger Chamber vs. Center Chamber, in LIT treated males	Stranger Chamber: *M* = 23.44, ±*SEM* = 11.89; Center Chamber: *M* = 4.69, ±*SEM* = 1.23 $U(N_{Stranger} = 6, N_{Center} = 6) = 7.0$, $p = 0.093$
S3F – Tx-4hr-PPT 90-180	Chamber Time	Mann-Whitney U test, comparison: Partner Chamber vs. Stranger Chamber, in LIT treated males	Partner Chamber: *M* = 60.87, ±*SEM* = 12.15; Stranger Chamber: *M* = 23.44, ±*SEM* = 11.89 $U(N_{Partner} = 6, N_{Stranger} = 6) = 7.0$, $p = 0.093$
S3G – Tx-4hr-PPT 0-90	Aggressive Events	Kruskal-Wallis test	$H(3) = 6.07$, $p = 0.10$
S3H – Tx-4hr-PPT 90-180	Aggressive Events	Kruskal-Wallis test	$H(3) = 3.61$, $p = 0.30$
S4A – Cohabitation	Huddle Time	Kruskal-Wallis test	$H(3) = 5.43$, $p = 0.14$
S4A – Cohabitation	Huddle Time	Mann-Whitney U test, comparison: VEH Treated vs. LIT Treated, During 0-90 of cohabitation	VEH Treated: *M* = 16.37, ±*SEM* = 10.62; LIT Treated: *M* = 17.94, ±*SEM* = 3.75 $U(N_{VEH} = 5, N_{LIT} = 4) = 7.0$, $p = 0.55$
S4A – Cohabitation	Huddle Time	Mann-Whitney U test, comparison: VEH Treated vs. LIT Treated, During 90-180 of cohabitation	VEH Treated: *M* = 32.63, ±*SEM* = 13.36; LIT Treated: *M* = 52.33, ±*SEM* = 7.96 $U(N_{VEH} = 5, N_{LIT} = 4) = 6.0$, $p = 0.41$
S4B – Cohabitation	Aggressive Events	Kruskal-Wallis test	$H(3) = 6.96$, $p = 0.06$
S4B – Cohabitation	Aggressive Events	Mann-Whitney U test, comparison: VEH Treated vs. LIT Treated, During 0-90 of cohabitation	VEH Treated: *M* = 24.8, ±*SEM* = 15.20; LIT Treated: *M* = 26.75, ±*SEM* = 12.75 $U(N_{VEH} = 5, N_{LIT} = 4) = 7.5$, $p = 0.59$
S4B – Cohabitation	Aggressive Events	Mann-Whitney U test, comparison: VEH Treated vs. LIT Treated, During 90-180 of cohabitation	VEH Treated: *M* = 23.0, ±*SEM* = 19.75; LIT Treated: *M* = 5.25, ±*SEM* = 5.25

			$U(N_{VEH} = 5, N_{LIT} = 4) = 8.5$, $p = 0.84$
S5A – 2hr-Tx-PPT 0-90	Huddle Time	Kruskal-Wallis test	$H(3) = 1.24$, $p = 0.74$
S5A – 2hr-Tx-PPT 0-90	Huddle Time	Mann-Whitney U test, comparison: Partner Animal vs. Stranger Animal, in VEH treated males	Partner Animal: $M = 20.49$, $\pm SEM = 5.40$; Stranger Animal: $M = 8.90$, $\pm SEM = 5.74$ $U(N_{Partner} = 5, N_{Stranger} = 5) = 8.0$, $p = 0.42$
S5A – 2hr-Tx-PPT 0-90	Huddle Time	Mann-Whitney U test, comparison: Partner Animal vs. Stranger Animal, in LIT-001 treated males	Partner Animal: $M = 10.92$, $\pm SEM = 6.61$; Stranger Animal: $M = 13.17$, $\pm SEM = 12.03$ $U(N_{Partner} = 5, N_{Stranger} = 5) = 11.0$, $p = 0.78$
S5B – 2hr-Tx-PPT 90-180	Huddle Time	Kruskal-Wallis test	$H(3) = 0.91$, $p = 0.82$
S5B – 2hr-Tx-PPT 90-180	Huddle Time	Mann-Whitney U test, comparison: Partner Animal vs. Stranger Animal, in VEH treated males	Partner Animal: $M = 33.07$, $\pm SEM = 19.45$; Stranger Animal: $M = 20.93$, $\pm SEM = 13.98$ $U(N_{Partner} = 5, N_{Stranger} = 5) = 8.0$, $p = 0.42$
S5B – 2hr-Tx-PPT 90-180	Huddle Time	Mann-Whitney U test, comparison: Partner Animal vs. Stranger Animal, in LIT-001 treated males	Partner Animal: $M = 10.76$, $\pm SEM = 7.41$; Stranger Animal: $M = 23.54$, $\pm SEM = 14.69$ $U(N_{Partner} = 5, N_{Stranger} = 5) = 11.0$, $p = 0.84$
S5C – Tx-4hr-PPT 0-90	Partner-Stranger Huddle Time, VEH treated males	One-Sample Wilcoxon Signed Rank Test	N = 5, Hypothesized med.= 0, W = 12.0, Z= 1.21, $p = 0.22$
S5C – Tx-4hr-PPT 0-90	Partner-Stranger Huddle Time, LIT treated males	One-Sample Wilcoxon Signed Rank Test	N = 5, Hypothesized med.= 0, W = 7.0, Z= -0.13, $p = 0.89$
S5D – Tx-4hr-PPT 90-180	Partner-Stranger Huddle Time, VEH treated males	One-Sample Wilcoxon Signed Rank Test	N = 5, Hypothesized med.= 0, W = 9.0, Z= 0.40, $p = 0.68$
S5D – Tx-4hr-PPT 90-180	Partner-Stranger Huddle Time, LIT treated males	One-Sample Wilcoxon Signed Rank Test	N = 5, Hypothesized med.= 0, W = 6.0, Z= -0.40, $p = 0.68$
S5E – 2hr-Tx-PPT 0-90	Chamber Time	Kruskal-Wallis test	$H(5) = 9.22$, $p = 0.10$
S5E – 2hr-Tx-PPT 0-90	Chamber Time	Mann-Whitney U test, comparison: Partner Chamber vs. Center Chamber, in VEH treated males	Partner Chamber: $M = 41.05$, $\pm SEM = 3.98$; Center Chamber: $M = 19.97$, $\pm SEM = 5.12$ $U(N_{Partner} = 5, N_{Center} = 5) = 2.0$, $p = 0.031$

S5E – 2hr-Tx-PPT 0-90	Chamber Time	Mann-Whitney U test, comparison: Stranger Chamber vs. Center Chamber, in VEH treated males	Stranger Chamber: M = 25.15, $\pm SEM$ = 7.51; Center Chamber: M = 19.97, $\pm SEM$ = 5.12 $U(N_{Stranger} = 5, N_{Center} = 5) = 10.0$, $p = 0.69$
S5E – 2hr-Tx-PPT 0-90	Chamber Time	Mann-Whitney U test, comparison: Partner Chamber vs. Stranger Chamber, in VEH treated males	Partner Chamber: M = 41.05, $\pm SEM$ = 3.98; Stranger Chamber: M = 19.97, $\pm SEM$ = 5.12 $U(N_{Partner} = 5, N_{Stranger} = 5) = 3.0$, $p = 0.055$
S5E – 2hr-Tx-PPT 0-90	Chamber Time	Mann-Whitney U test, comparison: Partner Chamber vs. Center Chamber, in LIT treated males	Partner Chamber: M = 35.64, $\pm SEM$ = 7.84; Center Chamber: M =, $\pm SEM$ = $U(N_{Partner} = 5, N_{Center} = 5) = 5.0$, $p = 0.15$
S5E – 2hr-Tx-PPT 0-90	Chamber Time	Mann-Whitney U test, comparison: Stranger Chamber vs. Center Chamber, in LIT treated males	Stranger Chamber: M = 35.63, $\pm SEM$ = 9.33; Center Chamber: M = 18.48, $\pm SEM$ = 5.61 $U(N_{Stranger} = 5, N_{Center} = 5) = 4.0$, $p = 0.095$
S5E – 2hr-Tx-PPT 0-90	Chamber Time	Mann-Whitney U test, comparison: Partner Chamber vs. Stranger Chamber, in LIT treated males	Partner Chamber: M = 35.64, $\pm SEM$ = 7.84; Stranger Chamber: M = 35.63, $\pm SEM$ = 9.33 $U(N_{Partner} = 5, N_{Stranger} = 5) = 12.0$, $p > 0.99$
S5F – 2hr-Tx-PPT 90-180	Chamber Time	Kruskal-Wallis test	$H(5) = 13.19$, $p = 0.021$
S5F – 2hr-Tx-PPT 90-180	Chamber Time	Mann-Whitney U test, comparison: Partner Chamber vs. Center Chamber, in VEH treated males	Partner Chamber: M = 38.44, $\pm SEM$ = 15.41; Center Chamber: M = 2.68, $\pm SEM$ = 1.20 $U(N_{Partner} = 5, N_{Center} = 5) = 1.0$, $p = 0.015$
S5F – 2hr-Tx-PPT 90-180	Chamber Time	Mann-Whitney U test, comparison: Stranger Chamber vs. Center Chamber, in VEH treated males	Stranger Chamber: M = 37.98, $\pm SEM$ = 15.19; Center Chamber: M = 2.68, $\pm SEM$ = 1.20 $U(N_{Stranger} = 5, N_{Center} = 5) = 8.0$, $p = 0.42$

S5F – 2hr-Tx-PPT 90-180	Chamber Time	Mann-Whitney U test, comparison: Partner Chamber vs. Stranger Chamber, in VEH treated males	Partner Chamber: M = 38.44, ±SEM = 15.41; Stranger Chamber: M = 37.98, ±SEM = 15.19 $U(N_{Partner} = 5, N_{Stranger} = 5) = 12.0$, $p > 0.99$
S5F – 2hr-Tx-PPT 90-180	Chamber Time	Mann-Whitney U test, comparison: Partner Chamber vs. Center Chamber, in LIT treated males	Partner Chamber: M = 25.64, ±SEM = 8.71; Center Chamber: M = 5.26, ±SEM = 1.48 $U(N_{Partner} = 5, N_{Center} = 5) = 3.0$, $p = 0.055$
S5F – 2hr-Tx-PPT 90-180	Chamber Time	Mann-Whitney U test, comparison: Stranger Chamber vs. Center Chamber, in LIT treated males	Stranger Chamber: M = 51.81, ±SEM = 10.70; Center Chamber: M = 5.26, ±SEM = 1.48 $U(N_{Stranger} = 5, N_{Center} = 5) = 0.0$, $p = 0.008$
S5F – 2hr-Tx-PPT 90-180	Chamber Time	Mann-Whitney U test, comparison: Partner Chamber vs. Stranger Chamber, in LIT treated males	Partner Chamber: M = 25.64, ±SEM = 8.71; Stranger Chamber: M = 51.81, ±SEM = 10.70 $U(N_{Partner} = 5, N_{Stranger} = 5) = 6.0$, $p = 0.22$
S5G – 2hr-Tx-PPT 0-90	Aggressive Events	Kruskal-Wallis test	$H(3) = 5.08$, $p = 0.16$
S5H – 2hr-Tx-PPT 90-180	Aggressive Events	Kruskal-Wallis test	$H(3) = 2.26$, $p = 0.51$
